# Compound 18 Improves Glucose Tolerance in a Hepatocyte TGR5-dependent Manner in Mice

**DOI:** 10.3390/nu12072124

**Published:** 2020-07-17

**Authors:** Marlena M. Holter, Margot K. Chirikjian, Daniel A. Briere, Adriano Maida, Kyle W. Sloop, Kristina Schoonjans, Bethany P. Cummings

**Affiliations:** 1 Department of Biomedical Sciences, College of Veterinary Medicine, Cornell University, Ithaca, NY 14850, USA; mmh277@cornell.edu (M.M.H.); mkc224@cornell.edu (M.K.C.); 2 Diabetes and Complications, Lilly Research Laboratories, Eli Lilly and Company, Indianapolis, IN 46225, USAsloop_kyle_w@lilly.com (K.W.S.); 3 Institute for Diabetes and Cancer, Helmholtz Zentrum München, 85764 Neuherberg, Germany; adriano.maida@helmholtz-muenchen.de (A.M.); krisitina.schoonjans@epfl.ch (K.S.); 4 Institute of Bioengineering, École Polytechnique Fédérale de Lausanne, 1015 Lausanne, Switzerland

**Keywords:** hepatocyte, TGR5, glucose regulation

## Abstract

The bile acid receptor, TGR5, is a key regulator of glucose homeostasis, but the mechanisms by which TGR5 signaling improves glucose regulation are incompletely defined. In particular, TGR5 has an increasingly appreciated role in liver physiology and pathobiology; however, whether TGR5 signaling within the liver contributes to its glucoregulatory effects is unknown. Therefore, we investigated the role of hepatocyte TGR5 signaling on glucose regulation using a hepatocyte-specific TGR5 knockout mouse model. Hepatocyte-specific *Tgr5^Hep+/+^* and *Tgr5^Hep−/−^* mice were fed a high fat diet (HFD) for 7 weeks and then orally gavaged with three doses of a highly potent, TGR5-specific agonist, Compound 18 (10 mg/kg), or vehicle, over 72 h and underwent an oral glucose tolerance test (OGTT) after the last dose. Herein, we report that TGR5 mRNA and protein is present in mouse hepatocytes. Cumulative food intake, body weight, and adiposity do not differ between *Tgr5^Hep+/+^* and *Tgr5^Hep−/−^* mice with or without treatment with Compound 18. However, administration of Compound 18 improves glucose tolerance in *Tgr5^HEP+/+^* mice, but not in *Tgr5^Hep−/−^* mice. Further, this effect occurred independent of body weight and GLP-1 secretion. Together, these data demonstrate that TGR5 is expressed in hepatocytes, where it functions as a key regulator of whole-body glucose homeostasis.

## 1. Introduction

Bile acids are amphipathic steroid molecules that activate the nuclear receptor, farnesoid X receptor (FXR), and the transmembrane G-protein coupled receptor, TGR5, to integrate lipid, glucose, and energy metabolism and maintain metabolic homeostasis [1,2,3]. Dysregulated bile acid signaling is associated with the pathogenesis of various diseases including cholestatic liver diseases, dyslipidemia, fatty liver diseases, and type 2 diabetes [4,5,6,7]. As such, TGR5 is a key regulator of metabolic homeostasis; however, the mechanisms remain incompletely defined.

TGR5 is expressed in many tissues including endocrine glands, adipocytes, muscle, liver, brain, and the gastrointestinal tract [8,9,10,11,12]. TGR5 signaling in many of these tissue types has been shown to contribute to glucose regulation [3,13]. For example, in gastrointestinal enteroendocrine L cells, TGR5 signaling promotes glucagon-like peptide-1 (GLP-1) secretion [3,14,15]. Furthermore, in mice fed a high fat diet (HFD), TGR5 agonists have been shown to increase in GLP-1 secretion from L cells, which was associated with an improvement in insulin sensitivity and measures of hepatic steatosis [16]. TGR5 is also found on pancreatic beta-cells where its activation increases glucose-stimulated insulin secretion [17]. It has also been demonstrated that TGR5 signaling increases energy expenditure by increasing the activity of the cyclic-AMP-dependent thyroid hormone activating enzyme type 2 iodothyronine deiodinase, mitochondrial thermogenesis, and fat mass oxidation to enhance basal metabolic rate [13,18,19]. TGR5 signaling in adipocytes promotes beiging of white adipose tissue in mice [19] and enhances energy expenditure to improve glucose regulation [13,20]. Finally, TGR5 signaling in immune cells decreases inflammatory cytokine secretion [11,21,22], which likely decreases systemic inflammation to improve insulin sensitivity [23].

TGR5 is also robustly expressed in the liver, but the role of hepatic TGR5 signaling in glucose regulation remains poorly understood. Various studies have identified anti-inflammatory, anti-apoptotic, choleretic, and proliferative effects of TGR5 signaling in nonparenchymal cell types of the liver. Within the liver, TGR5 is highly expressed on Kupffer cells [9], sinusoidal endothelial cells [10], and cholangiocytes [24,25]. In Kupffer cells and resident macrophages, bile acid signaling through TGR5 activates a cAMP-dependent pathway that attenuates LPS-induced cytokine expression and reduces the NF-kB-dependent inflammatory response, thereby dampening hepatic inflammation and promoting tissue remodeling [9,21]. Further, activation of TGR5 on sinusoidal endothelial cells functions to modulate liver microcirculation through increased production of nitric oxide [10,26]. This serves to both mitigate portal hypertension and enable adaptation of hepatic blood flow to nutrient uptake [26,27]. TGR5 activation of biliary epithelial cells results in CFTR-dependent chloride and bicarbonate secretion into bile, which reduces bile acid protonation to protect the liver parenchyma from bile acid toxicity [25,28,29,30]. Additionally, TGR5 stimulates relaxation of gallbladder smooth muscle cells to induce gallbladder filling [31,32]. TGR5 has also been shown to induce cholangiocyte proliferation [33] and promote barrier function by reinforcing cholangiocyte tight junctions [30].

Previous studies highlight a role for TGR5 in liver biology and pathobiology. For example, the genetic ablation of TGR5 or the inactivation of TGR5 signaling has been shown to make mice more susceptible of cholestatic liver injuries [30,33,34,35]. This is thought to be due to a role for TGR5 in maintaining a healthy bile acid profile. Specifically, mice with homozygous deficiency of TGR5 exhibit decreased total bile acid pool size [8,24,32], an excessively hydrophobic bile acid pool [32,36], as well as protection from gallstone formation when fed a lithogenic diet [24]. In addition, treatment of HFD-fed mice with a TGR5-specific agonist, INT-777, decreased liver steatosis [3,24], suggesting that TGR5 signaling in the liver attenuates triglyceride accumulation. Furthermore, HFD-fed whole body *Tgr5*^−/−^ mice are more susceptible to liver injury than littermate controls as evidenced by an elevation of serum liver enzymes due to increased cytokine mRNA levels, more pronounced inflammatory infiltrates, and increased liver necrosis [34,37], highlighting the hepatoprotective role of TGR5 signaling. Together, these data demonstrate that TGR5 exerts important effects on various aspects of liver health. As the liver is a key organ involved in whole body glucose regulation, this suggests that liver TGR5 signaling may be a critical contributor to the overall metabolic benefits of TGR5 agonists.

Despite the growing body of literature regarding the role of TGR5 in liver health, it is unknown if TGR5 signaling within the liver contributes to TGR5′s role in glucose regulation, largely due to a lack of cell-type specific in vivo studies of liver TGR5 function. As the hepatocyte is the predominant cell type in the liver, here, we tested the hypothesis that TGR5 signaling in the hepatocyte improves glucose regulation. It is thought that TGR5 is, at most, lowly expressed on hepatocytes; however, this has not been extensively studied. TGR5 expression has been identified in a human hepatocellular carcinoma cell line [38], in canine hepatocytes [39], and here, in mouse hepatocytes. In this study, we employed a highly potent and specific non-bile acid TGR5 agonist, Compound 18 [40], as well as hepatocyte-specific TGR5 knockout mice to investigate the role of hepatocyte TGR5 on glucose regulation. Our results demonstrate that Compound 18 enhances glucose regulation in a hepatocyte TGR5-dependent manner.

## 2. Materials and Methods

### 2.1. Animals and Diet

All experiments were performed in accordance with the Guide for the Care and Use of Laboratory Animals and approved by the Institutional Animal Care and Use Committee of Cornell University (approved animal protocol number: 2013-0065). Study mice were individually housed and maintained in a temperature and humidity-controlled room, with a 14:10 h light-dark cycle. Whole body TGR5 knockout mice (B6.Gpbar1 ^tm1(KOMP)Vlcg^) (KOMP Repository, The Knockout Mouse Project; University of California, Davis, CA, USA) were used for immunofluorescence analysis of hepatocyte TGR5 expression. Hepatocyte-specific TGR5 knockout mice were generated by crossing a TGR5 floxed mouse line (B6.Gpbar1 ^< tm1.1Auw^/J) [23] with hepatocyte-specific albumin-Cre mouse line (B6N.Cg-Tg (Alb-cre)^21Mgn^/J). To validate this model, hepatocytes were isolated from *Tgr5^HEP+/+^* and *Tgr5^HEP^*^−/−^ mice and analyzed by RT-PCR to confirm the presence of *Tgr5* mRNA in *Tgr5^HEP+/+^* mice and loss of *Tgr5* mRNA in the hepatocytes of *Tgr5^HEP^*^−/−^ mice (Figure S1). Starting at 8 weeks of age, male and female *Tgr5^HEP+/+^* and *Tgr5^HEP^*^−/−^ littermates were fed a HFD consisting of ground chow (5012 LabDiets; St. Louis, MO, USA) supplemented with 3.4% butter fat, 8.5% tallow, 13.1% soybean oil, 3.5% mineral mix, and 1% vitamin mix (Dyets; Bethlehem, PA, USA) by weight for 7 weeks to produce an obese, insulin resistant phenotype. Mice were matched for baseline body weight at the start of the HFD. Food intake and body weight were measured once per week (*Tgr5^HEP+/+^ n* = 16, 8 males, 8 females; *Tgr5^HEP^*^−/−^; *n* = 12; 7 males, 5 females). In a separate cohort of mice, following 7 weeks of the HFD, mice received 3 consecutive, daily doses of either vehicle (20% Captisol *w*/*v* with water, CyDex Pharmaceuticals) or Compound 18 (10 mg/kg/day, Eli Lilly & Company-molecular weight = 508.62) by oral gavage. Compound 18 was formulated in 20% Captisol w/v with water, as previously described [40]. An oral glucose tolerance test (OGTT, 2 g/kg body weight oral gavage with dextrose), following an overnight (12 h) fast, was performed as previously described [41]. To minimize the contribution of TGR5-stimulated GLP-1 release, the OGTT was performed 1 h after the last dose of Compound 18 [40]. Blood glucose measurements were made using a glucometer (One-Touch Ultra, Lifescan; Milpitas, CA, USA). Serum insulin concentrations were measured by ELISA (Millipore; Burlington, MA, USA) and serum total GLP-1 concentrations were measured by sandwich electrochemiluminescence immunoassay (Meso Scale Discovery; Gaithersburg, MA, USA). Immediately following the OGTT, mice were euthanized by an overdose of pentobarbital (200 mg/kg i.p.) and tissues were weighed and collected. The following groups were studied: Vehicle *Tgr5^HEP+/+^* (VEH *Tgr5^HEP+/+^*; *n* = 9; 4 males, 5 females), Compound 18 *Tgr5^HEP+/+^* (C18 *Tgr5^HEP+/+^*; *n* = 8; 4 males, 4 females), Vehicle *Tgr5^HEP^*^−/−^ (VEH *Tgr5^HEP^*^−/−^; *n* = 9; 4 males, 5 females), and Compound 18 *Tgr5^HEP^*^−/−^ (C18 *Tgr5^HEP^*^−/−^; *n* = 9; 4 males, 5 females).

### 2.2. HOMA-IR Calculation

The HOMA-IR (homeostasis model assessment of insulin resistance) index was calculated as (fasting serum glucose × fasting serum insulin/22.5) to assess insulin resistance [42]. Log (HOMA-IR) was used as a surrogate index of insulin resistance, which has been validated for use in rodents, as previously described [43].

### 2.3. Immunofluorescence

**Figure 1:**
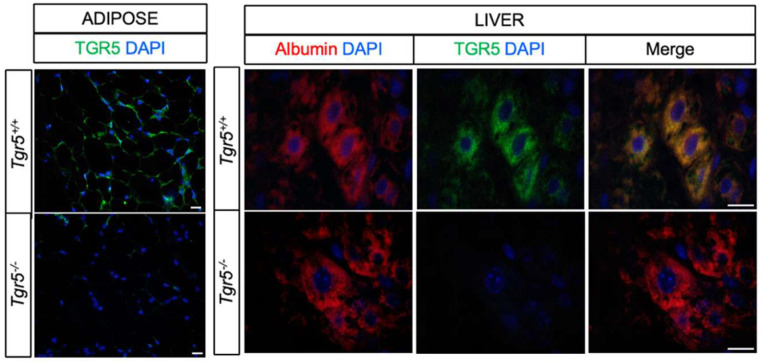
TGR5 is expressed in hepatocytes. Representative images of whole body TGR5 wild-type (*Tgr5^+/+^*) and knockout (*Tgr5^−/−^*) mouse adipose and liver sections immunostained for TGR5 (green), albumin (red), and DAPI. Scale bar = 20 um.

Liver samples from whole body TGR5 wildtype (*Tgr5*^+/+^) and knockout (*Tgr5*^−/−^) mice were used for immunofluorescence analysis, as previously described [41]. Briefly, samples were collected, fixed in 4% paraformaldehyde, and paraffin embedded. Sections were deparafinized in a xylene ethanol series, placed in Tris-EDTA buffer for antigen retrieval (10 mM Tris, 1 mM EDTA, 0.05% Tween, pH = 9.0), and then blocked in 5% bovine serum albumin. Sections were immunostained for TGR5 using a polyclonal anti-rabbit antibody (LSBio; Seattle, WA, USA; 1:500) and for albumin using a monoclonal anti-mouse antibody (Santa Cruz Biotechnology; Dallas, TX, USA; 1:500). The antibody against TGR5 was validated on adipose samples from whole-body TGR5 wildtype (*Tgr5*^+/+^) and knockout (*Tgr5*^−/−^) (Figure 1). Detection of the primary antibodies was performed using Alexa Flour 488 anti-rabbit and Alexa Fluor 633 anti-mouse secondary antibodies (1:500) (Invitrogen; Foster City, CA, USA). Nuclei were detected using 4′,6′-diamino-2-phenyl inodole (DAPI), which was included in the mounting solution (Invitrogen; Foster City, CA, USA). Images were captured using Nikon Eclipse E400 fluorescent microscope with Olympus DP73 color camera (final magnification 20× for adipose and 100× for liver).

### 2.4. Statistics and Data Analysis

Data are presented as mean ± SEM. The main effect of sex was not significant so the data for males and females were combined. All statistical analyses were performed using GraphPad Prism 8.00 for Mac (GraphPad Software, San Diego, CA, USA). Data were analyzed by two-factor ANOVA with Bonferroni’s post-test or Student’s *t*-test, as indicated. Differences were considered significant at *p* < 0.05.

## 3. Results

### 3.1. TGR5 Is Expressed in Hepatocytes

TGR5 is highly expressed in the liver [9,10,24,25]; however, whether TGR5 is expressed in mouse hepatocytes has not been previously reported. Therefore, we assessed TGR5 expression in mouse liver sections. Adipose tissue sections from a whole body TGR5 knockout mouse model were used for antibody validation (Figure 1). As previously reported [11,44], TGR5 was highly expressed in adipocytes. TGR5 was not detected in adipocytes from *Tgr5^−/−^* mice, confirming antibody specificity. TGR5 expression was also detected in some, but not all, hepatocytes in *Tgr5^+/+^*, but not *Tgr5^−/−^* mice (Figure 1). These data are the first to demonstrate that while lowly expressed, TGR5 is present in mouse hepatocytes. Given that hepatocytes comprise the majority of the liver parenchyma and are a key determinant of whole body glucose homeostasis, we used a hepatocyte-specific TGR5 knockout mouse model to determine the role of hepatocyte TGR5 signaling in metabolic health.

### 3.2. Hepatocyte TGR5 Does Not Contribute to Regulation of Food Intake, Body Weight, or Adiposity

**Figure 2:**
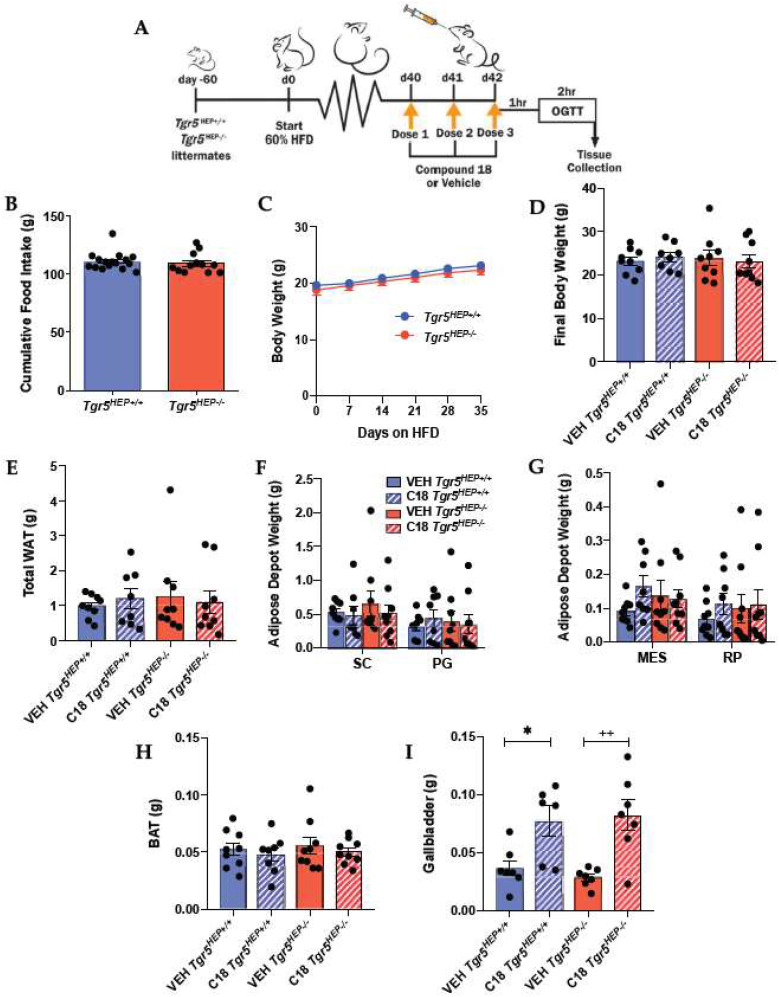
Hepatocyte TGR5 does not contribute to regulation of food intake, body weight, or adiposity. (**A**) Study design. (**B**) Cumulative food intake and (**C**) body weight over 7 weeks of high fat diet (HFD) feeding in *Tgr5^HEP+/+^* and *Tgr5^HEP^*^−/−^ mice. *n* = 12–16. (**D**) Body weight at the time of euthanasia; (**E**) total white adipose tissue (WAT) weight; (**F**) subcutaneous (SC) and perigonadal (PG); (**G**) mesenteric (MES) and retroperitoneal (RP) adipose tissue weights; (**H**) brown adipose tissue (BAT) weights; and (**I**) gallbladder weight in *Tgr5^HEP+/+^* and *Tgr5^HEP^*^−/−^ mice treated with Compound 18 (C18) or vehicle (VEH). *n* = 6–9. * *p* < 0.05 compared with VEH *Tgr5^HEP+/+^*; ^++^ *p* < 0.01 compared with VEH *Tgr5^HEP^*^−/−^ by two-factor ANOVA.

To assess the role of hepatocyte TGR5 on the regulation of body weight, we measured body weight and food intake in *Tgr5^HEP+/+^* and *Tgr5^HEP^*^−/−^ mice over the course of 7 weeks of HFD (Figure 2A). Similar to previous work in whole body TGR5 knockout mouse models [24,41,45], cumulative food intake and body weight did not differ between *Tgr5^HEP+/+^* and *Tgr5^HEP−/−^* mice (Figure 2B,C), which allowed us to assess the body weight-independent effects of hepatocyte TGR5 signaling on glucose regulation. In addition, final body weight and adiposity, measured after 3 consecutive daily doses of Compound 18 or vehicle, did not differ between genotype or treatment (Figure 2D–H). These data demonstrate that hepatocyte TGR5 does not regulate food intake, body weight, or adiposity under basal conditions or following stimulation by Compound 18.

TGR5 is highly enriched in the biliary tract [24,25] and its absence has been shown to slow bile flow and reduce gallbladder volume [31,32]. Consistent with this, Compound 18 and other synthetic TGR5 agonists have been reported to increase gallbladder filling [40,46,47]. Therefore, we assessed gallbladder weight at the time of euthanasia in a sub-set of mice to determine the impact of Compound 18 treatment on this negative side-effect. Treatment with Compound 18 increased gallbladder weight in both *Tgr5^HEP+/+^* and *Tgr5^HEP−/−^* mice (Figure 2I, *p* < 0.05). These data demonstrate that Compound 18 promotes gallbladder filling independently of hepatocyte TGR5 signaling.

### 3.3. Compound 18 Improves Glucose Regulation in a Hepatocyte TGR5-dependent Manner

**Figure 3:**
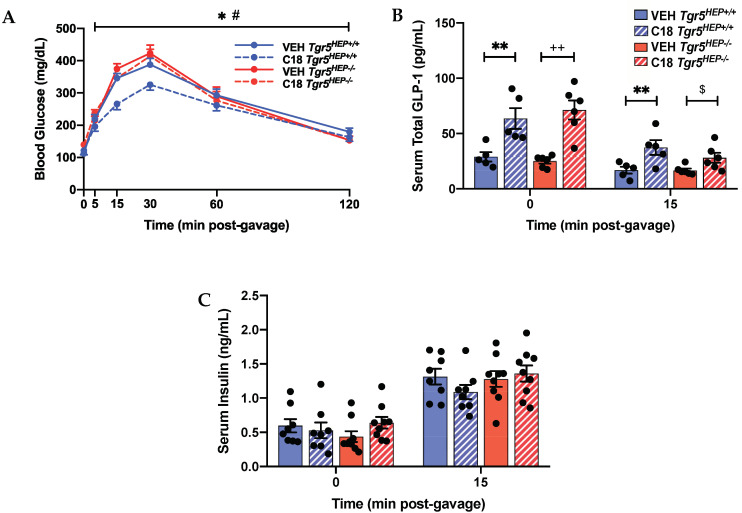
Compound 18 improves glucose regulation in a hepatocyte TGR5-dependent manner. (**A**) Blood glucose, (**B**) serum total GLP-1, and (**C**) serum insulin concentrations during an OGTT in *Tgr5^HEP+/+^* and *Tgr5^HEP^*^−/−^ mice after 3 doses of Compound 18 (C18) or vehicle (VEH). *n* = 5–9. * *p* < 0.05, ** *p* < 0.01 C18 *Tgr5^HEP+/+^* vs. VEH *Tgr5^HEP+/+^*; ^++^ *p* < 0.01 C18 *Tgr5^HEP^*^−/−^ vs. VEH *Tgr5^HEP^*^−/−^; ^#^ *p* < 0.05 C18 *Tgr5^HEP^*^−/−^ vs. C18 *Tgr5^HEP+/+^* by two-factor ANOVA; ^$^ *p* < 0.05 compared with VEH *Tgr5^HEP−/−^* by Student’s *t*-test.

To assess the role of hepatocyte TGR5 on glucose regulation, we performed an OGTT in HFD-fed *Tgr5^HEP+/+^* and *Tgr5^HEP^*^−/−^ mice with and without Compound 18 treatment. Compound 18 improved glucose tolerance compared with vehicle-treated controls in *Tgr5^HEP+/+^* mice, but not *Tgr5^HEP−/−^* mice (Figure 3A, *p* < 0.05). Furthermore, Compound 18-treated *Tgr5^HEP+/+^* mice exhibited lower blood glucose excursions compared with Compound 18-treated *Tgr5^HEP−/−^* mice (Figure 3A, *p* < 0.05).

A key mechanism by which TGR5 agonists improve glucose tolerance is through induction of GLP-1 secretion and subsequent enhancement of glucose-stimulated insulin secretion [3]. Previous work finds that while Compound 18 potently promotes GLP-1 secretion, GLP-1 levels return to baseline within approximately 1 h of Compound 18 administration in mice [40]. Therefore, to control for the effect of GLP-1, we performed the OGTT 1 h following the last dose of Compound 18 or vehicle. As baseline fasting blood samples were collected approximately 45 min after the last Compound 18 dose, fasting total serum GLP-1 levels were still elevated in Compound 18-treated *Tgr5^HEP+/+^* and *Tgr5^HEP^*^−/−^ mice compared to vehicle controls (Figure 3B, *p* < 0.05). While this Compound 18-induced increase in serum GLP-1 levels was diminished by 15 min post-glucose gavage, there remained an elevation of serum GLP-1 levels in Compound 18-treated mice compared with control (*p* < 0.05 by 2-factor ANOVA in the *Tgr5^HEP+/+^* mice and *p* < 0.05 by Student’s *t*-test in the *Tgr5^HEP−/−^* mice). Nevertheless, fasting serum insulin concentrations and serum insulin concentrations at 15 min after the glucose gavage did not differ between the genotype or treatment condition (Figure 3C). As expected, serum insulin levels at 15 min post-glucose gavage were elevated compared with fasting serum insulin concentrations in all groups. Of note, the lack of a difference in fasting serum insulin concentrations despite marked elevations in fasting serum GLP-1 concentrations points to the glucose-dependent actions of GLP-1 to promote insulin secretion. Together, these data demonstrate that hepatocyte TGR5 signaling contributes to the effect of Compound 18 to improve glucose tolerance, independently of insulin and GLP-1 secretion.

### 3.4. An Index of Insulin Sensitivity Is Impaired in Compound 18-treated Mice Lacking Hepatocyte TGR5

As expected, we did not observe alterations in insulin secretion during the OGTT between genotype or between treatment. It has been previously shown that enhanced TGR5 signaling improves insulin resistance in various tissues by decreasing inflammation [23] and lipotoxicity [16] and increasing energy expenditure [13,20], but the hepatocyte-specific effect of TGR5 signaling on insulin resistance has not been investigated. Therefore, we evaluated an index of insulin resistance in *Tgr5^HEP+/+^* and *Tgr5^HEP^*^−/−^ mice with and without Compound 18 treatment. We chose the log(HOMA-IR) as a surrogate index of insulin sensitivity as it has been shown to have improved predictive accuracy compared with other indices [43].

**Figure 4:**
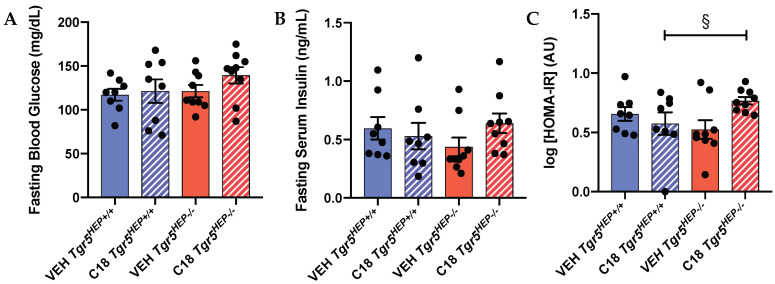
Compound 18 improves insulin sensitivity in a hepatocyte TGR5-dependent manner. (**A**) Fasting blood glucose, (**B**) fasting serum insulin concentrations, and (**C**) log(HOMA-IR) in *Tgr5^HEP+/+^* and *Tgr5^HEP^*^−/−^ mice after 3 doses of Compound 18 (C18) or vehicle (VEH). *n* = 8–9. ^§^ *p <* 0.05 compared with Compound 18 *Tgr5^HEP+/+^* by one-tailed Student’s *t*-test.

There was no significant difference in fasting blood glucose (Figure 4A) or fasting serum insulin concentrations (Figure 4B) between genotype or treatment. However, Compound 18-treated *Tgr5^HEP+/+^* mice exhibited a lower log(HOMA-IR) compared to Compound 18-treated *Tgr5^HEP−/−^* mice (Figure 4C, *p* < 0.05 by Student’s *t*-test). Together, with a lack of a difference in glucose-stimulated insulin secretion, these data suggest that hepatocyte TGR5 signaling may improve glucose homeostasis, in part, through an improvement in insulin sensitivity. Nevertheless, further work is needed to define the impact of hepatocyte TGR5 signaling on insulin sensitivity.

## 4. Discussion

The TGR5 receptor is ubiquitously expressed throughout the body and has a well-defined role in many regulatory functions that affect hepatic metabolism and extrahepatic glucose homeostasis [3,9,13,21]; however, the expression of TGR5 and its functional significance in hepatocytes remained unknown to date. In this study, we performed the first targeted assessment of TGR5 expression in mouse hepatocytes. TGR5 was detected in hepatocytes of paraffin embedded liver sections by immunofluorescence staining. Our understanding of the liver-specific effects of TGR5 on glucose regulation remain limited due to a lack of cell-type specific in vivo studies of liver TGR5 function. To this end, we employed a hepatocyte-specific TGR5 knockout mouse model in order to dissect the functional significance of hepatocyte TGR5 signaling. Herein, we provide compelling data that TGR5 is expressed in hepatocytes, where it functions as a regulator of whole body glucose homeostasis.

TGR5 has been implicated in body weight regulation through its role in energy metabolism. Specifically, TGR5 regulates energy expenditure by inducing mitochondrial biogenesis and thereby increasing basal metabolism in thermogenically competent tissues, such as brown adipose tissue and skeletal muscle and through the beiging of white adipose tissue [13,18,19,48]. In this study, there was no difference in body weight or adiposity between *Tgr5^HEP+/+^* and *Tgr5^HEP^*^−/−^ mice with or without treatment with Compound 18, demonstrating that hepatocyte TGR5 signaling does not regulate body weight, food intake, or adiposity. While Briere et al. reported that 14 days of Compound 18 administration at 60 mg/kg reduced body weight and fat mass gain in HFD-fed mice [40], it is likely that our 3-day dosing paradigm at 10 mg/kg was not long enough or robust enough to induce Compound 18-dependent reductions in adiposity. Nevertheless, the absence of genotype-dependent and Compound 18-dependent effects on adiposity and body weight in this study enabled us to assess the body weight-independent effects of hepatocyte TGR5 signaling on glucose homeostasis.

While our data are consistent with previous work demonstrating that TGR5 ablation contributes to metabolic dysregulation in mouse models [3,19,34,37,49], our data are the first to demonstrate that TGR5 signaling specifically within the hepatocyte plays a significant role in whole body glucose regulation. Until now, the role of TGR5 in the maintenance of glucose homeostasis and insulin sensitivity has been attributed to its effects on mitochondrial function in muscle and BAT and/or insulin release from the pancreas, enhanced by enteroendocrine L cell GLP-1 secretion [3,13,14,15,17,20]. For example, various TGR5 agonists have been shown to ameliorate glucose intolerance in obese and diabetic mice [3,13,50,51]. While in some studies, this improvement in glucose tolerance was associated with increased energy expenditure and subsequent weight loss [3,50], other studies attributed body weight-independent improvements in hyperglycemia to enhanced GLP-1 secretion [40,52]. In contrast, our data show that Compound 18 improves glucose tolerance in a hepatocyte TGR5-dependent manner. Further, in contrast to the aforementioned studies, this effect can occur independently of body weight, GLP-1 secretion, and glucose-stimulated insulin secretion. Thus, we provide evidence that TGR5 agonists improve glucose homeostasis through an additional novel mechanism specific to hepatocyte TGR5 signaling.

The hepatocyte TGR5-dependent improvement in glucose tolerance occurred in the absence of a difference in insulin secretion, suggesting that hepatocyte TGR5 signaling regulates hepatic glucose metabolism and/or insulin sensitivity. Hepatic glucose metabolism is dictated predominantly by hepatic glucose output and insulin sensitivity [53]. Using an index of insulin resistance, our data suggest that hepatocyte TGR5 signaling may regulate insulin sensitivity. Recent studies have proposed that HOMA-IR measurements of insulin resistance refer mostly to the liver, rather than describing peripheral insulin sensitivity [54,55]. This is of particular interest in our model as we sought to understand how liver TGR5 regulates glucose homeostasis. Consistent with our findings, previous work has shown that administration of INT-777 reduced insulin resistance in liver and muscle in obese mice, as assessed by a hyperinsulinemic euglycemic clamp and ^14^C-2-deoxy-glucose tracers [3]. It is also possible that the improved hepatic insulin resistance in *Tgr5^HEP+/+^* mice, as compared to *Tgr5^HEP^*^−/−^ mice, following treatment with Compound 18 is mediated by reduced inflammation and decreased ectopic triglyceride deposition, characteristic of enhanced liver TGR5 signaling [3,24]. Nevertheless, further work is needed to determine the mechanisms by which hepatocyte TGR5 signaling improves glucose tolerance.

A growing body of literature has highlighted the potential value of TGR5 agonists in the treatment of various metabolic and liver diseases [3,13,20,23,40,41,52]. However, the clinical development of TGR5 agonists is complicated by the wide range of effects associated with systemic TGR5 activation. TGR5 expression in mouse and human gallbladder is estimated to be 10-fold higher than any other tissue [24,25,40]. Studies in whole body *Tgr5^−/−^* mice have documented decreased bile flow from the liver [32] and reduced gallbladder volume [31,32], which is explained by reduced TGR5-dependent biliary secretion and impaired smooth muscle relaxation of the gallbladder, respectively. In contrast, the oral administration of TGR5 agonists, including INT-777, oleanolic acid, Compound 23g, and RO552739, has been shown to induce hepatic bile flow [32,52] and increase gallbladder filling in wild type mice [32,46,47,50]. Similarly, Compound 18 has been shown to have a dose-dependent effect on gallbladder filling, an effect that is lost in whole body *Tgr5^−/−^* mice [40]. To this end, a major side effect of TGR5 agonists is the inhibition of gallbladder emptying, ultimately leading to cholestatic liver injury [24,32,40]. As expected, in this present study, we found no difference in gallbladder weight between *Tgr5^HEP+/+^* and *Tgr5^HEP^*^−/−^ mice in the absence of Compound 18. However, in response to Compound 18 administration, our data demonstrate a significant increase in gallbladder weight in both *Tgr5^HEP+/+^* and *Tgr5^HEP^*^−/−^ mice. Together, these results suggest that hepatocyte TGR5 does not contribute to the role of TGR5 in gallbladder filling under basal conditions or in the presence of a TGR5 agonist. Further, our results highlight the need to identify cell-type specific effects and downstream signaling targets of TGR5 signaling in order to develop better bile acid-based therapeutics to treat diabetes and metabolic disease.

## 5. Conclusions

In conclusion, TGR5 is a promising therapeutic target, but its breadth of actions, particularly in the gallbladder, complicates this task. As such, there is a growing need to identify cell-type specific effects of TGR5 signaling in order to begin to identify and target the downstream effectors of TGR5 signaling. Our findings provide additional insight into the underlying mechanisms by which TGR5 agonists improve whole body glucose homeostasis. Importantly, our data demonstrate that hepatocyte TGR5 signaling regulates whole body glucose homeostasis.

## Supplementary Materials

The following are available online at https://www.mdpi.com/2072-6643/12/7/2124/s1, Figure S1: Validation of hepatocyte-specific TGR5 knockout mouse model.
