# Supplementary material for: Compound 18 Improves Glucose Tolerance in a Hepatocyte TGR5-dependent Manner in Mice
Source: Nutrients. 2020 Jul 17;12(7):2124. doi: 10.3390/nu12072124 (PMC7400836; doi:10.3390/nu12072124)
Supplement: Supplementary file 1 [file nutrients-12-02124-s001.pdf]

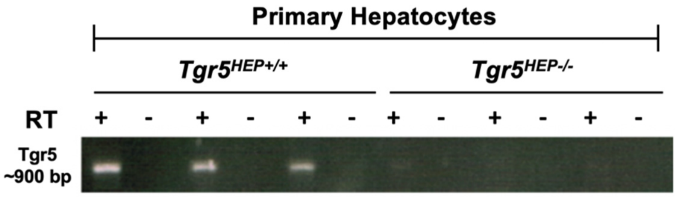

Supplemental Figure 1. Validation of hepatocyte-specific TGR5 knockout mouse model. RT-PCR for *Tgr5* expression in hepatocytes isolated from *Tgr5*$^{HEP+/+}$ and *Tgr5*$^{HEP-/-}$ mice. *n*=3 per group. (+) indicates that the RT enzyme was present during the RT reaction; (-) indicates that the RT enzyme was not present during the RT reaction.
